# *Janthinobacterium tructae* sp. nov., Isolated from Kidney of Rainbow Trout (*Oncorhynchus mykiss*)

**DOI:** 10.3390/pathogens10020229

**Published:** 2021-02-19

**Authors:** Won Joon Jung, Sang Wha Kim, Sib Sankar Giri, Hyoun Joong Kim, Sang Guen Kim, Jeong Woo Kang, Jun Kwon, Sung Bin Lee, Woo Taek Oh, Jin Woo Jun, Se Chang Park

**Affiliations:** 1 Laboratory of Aquatic Biomedicine, College of Veterinary Medicine and Research Institute for Veterinary Science, Seoul National University, Seoul 08826, Korea; cwj0125@snu.ac.kr (W.J.J.); blackcat9201@snu.ac.kr (S.W.K.); ssgiri@snu.ac.kr (S.S.G.); hjoong1@nate.com (H.J.K.); imagine0518@snu.ac.kr (S.G.K.); kck90victory@naver.com (J.W.K.); kjun1002@naver.com (J.K.); lsbin1129@naver.com (S.B.L.); mike0202@snu.ac.kr (W.T.O.); 2 Department of Aquaculture, Korea National College of Agriculture and Fisheries, Jeonju 54874, Korea

**Keywords:** *Jantinobacterium*, *Jantinobacterium tructae*, rainbow trout, *Oncorhynchus mykiss*

## Abstract

This study presents a novel *Janthinobacterium* strain, SNU WT3, isolated from the kidney of rainbow trout. A phylogenetic study using 16S rRNA sequences indicated that the strain is closely related to *Janthinobacterium svalbardensis* JA-1^T^. However, biochemical analysis found differences in D-xylose adonitol, N-acetylglucosamine, arbutin, and cellobiose. As for genome-to-genome distance and average nucleotide identity values calculated between strain SNU WT3 and other related strains such as *J. lividum* EIF1, *J. svalbardensis* PAMC 27463, and *J. agaricidamnosum* BHSEK were all below the cutoff value between species. DNA-DNA hybridization between strain SNU WT3 and other close relatives indicated the results of *J. lividum* DSM 1522^T^ (47.11%) and *J. svalbardensis* JA-1^T^ (38.88%) individually. The major fatty acid compositions of strain SNU WT3 were cylco-C_17:0_ (41.45%), C_16:0_ (33.86%) and C_12:0_ (5.87%). The major polar lipids were phosphatidylethanolamine, phosphatidylcholine, phosphatidylglycerol, and diphosphatidylglycerol. The quinone system was composed mainly of ubiquinone Q-8. The genome of strain SNU WT3 consists of 6,314,370 bp with a G + C content of 62.35%. Here, we describe a novel species of the genus *Janthinobacterium*, and the name *Janthinobacterium tructae* has been proposed with SNU WT3^T^ (=KCTC 72518 = JCM 33613) as the type strain.

## 1. Introduction

Members of the genus *Janthinobacterium* are Gram-negative, rod-shaped, psychrotolerant bacteria normally found in environments such as soil, water, and the Arctic glaciers [1,2]. *Janthinobacterium lividum,* one of the earliest discovered *Janthinobacterium* species, was reported in 1978, and the species is found frequently in our nearest environment, including green onions, salad mix, water, and soil [3,4]. Since the discovery of *J. lividum,* only a few more *Janthinobacterium* species have been reported. *Jantinobacterium agaricidamnosum* was isolated in 1999 and is known to cause soft rot disease of the mushroom *Agaricus bisoporus* [5]. *Janthinobacterium svalbardensis* was identified in 2013, isolated from ice samples of the island Spitsbergen in the Arctic [6]. *Janthinobacterium psychrotolerans* S3-2^T^, isolated from a freshwater pond near Aarhus, Denmark, was described in 2017 [7]. Three more species were described in 2020 from tropical and subtropical rivers of China: *Janthinobacterium violaceinigrum* FT13W^T^, *Janthinobacterium aquaticum* FT58W^T^, and *Janthinobacterium rivuli* FT68W^T^ [8]. Only a few studies have described *Janthinobacterium* species as a pathogen. The species was considered nonpathogenic to humans until the first report of septicemia in Thailand in 1992 [9]. Some studies also describe *Janthinobacterium* as a fish pathogen, mostly affecting the rainbow trout (*Oncorhynchus mykiss*) [10,11].

Since not many *Janthinobacterium* species have been identified, only a few studies have been published about them. Despite this, the violacein complex of the *Janthinobacterium* that consists of the genes *vioA*, *vioB*, *vioC*, *vioD*, and *vioE* has been the focus of considerable research [12,13]. *J. lividum* is well known for producing pigment [2,14]. As the compound violacein, which induces purple pigmentation, has distinctive antiviral, antibacterial, and antifungal properties, *J. lividum* has been used for the treatment of *Batrachochytrium dendrobatidis* infection in amphibians [15]. Among the strains which were described in 2020, *Janthinobacterium violaceinigrum* FT13W^T^ and *Janthinobacterium rivuli* FT68W^T^ produce violacein [8]. However, not all Janthinobacteria produce violacein. *J. agaricidamnosum* is a non-pigmented soft rot pathogen [5]. *J. svalbardensis* produces dark red-brown to black pigmentation, which was designated as a violacein-like pigment, different from the purple pigment of *J. lividum* [6]. *J. psychrotolerans* S3-2^T^ lacks the ability to produce violacein but shows antibiotic resistance, incomplete denitrification, and fermentation [7].

In this study, we isolated a novel pathogen, SNU WT3, from the swollen kidney of diseased rainbow trout (*Oncorhynchus mykiss*) that exhibited abnormal swimming behavior from a farm in the Republic of Korea. The identification of the bacterium was performed using 16S rRNA gene-based phylogenetic analysis, biochemical analysis, chemotaxonomic characteristics, and complete genome analysis. We present the genome of strain SNU WT3 as well as its phenotypic features and classification.

## 2. Results

### 2.1. Bacterial Characteristics

#### 2.1.1. Histopathology

**Figure 1:**
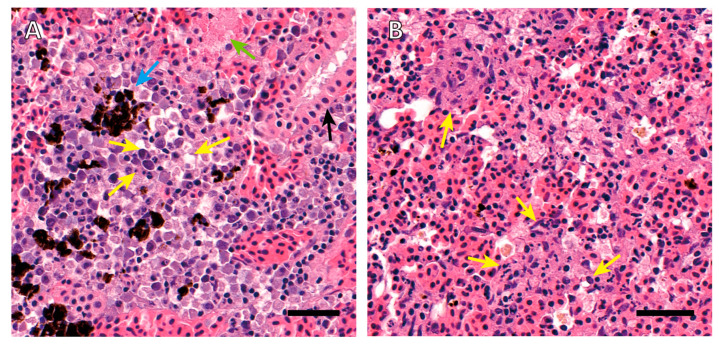
Histopathology of kidney and spleen of a moribund rainbow trout with *Janthinobacterium tructae* strain SNU WT3 infection. (**A**) Histopathology of the kidney showed hyperemia and melanomacrophage accumulation in the interstitium (blue arrow). Karyolysis, pyknosis, karyorrhexis, and hydropic and vacuolar degeneration of interstitial hematopoietic cells can also be observed (yellow arrows). Epithelial cell pyknosis (black arrow) and eosinophilic droplet accumulation (green arrow) existed in renal tubules. (**B**) Histopathology of the spleen showed a wide range of necrotizing hematopoietic cells with eosinophilic color change. Karyolysis, pyknosis, and karyorrhexis can also be observed (yellow arrows). Slides were stained with hematoxylin and eosin. Scale bars = 40 μm.

Intensive necrotic changes in hematopoietic tissues of the kidney and spleen were observed. Karyolysis, pyknosis, karyorrhexis, and hydropic and vacuolar degeneration occurred to interstitial hematopoietic cells of the kidney (Figure 1A). In renal tubules, epithelial cell pyknosis and eosinophilic droplet accumulation existed. Hyperemia and melanomacrophage accumulation in the interstitium could also be observed. Hematopoietic tissue of the spleen showed eosinophilic staining and the features of karyorrhexis, karyolysis, and pyknosis, indicating a wide range of tissues are in the process of necrosis (Figure 1B).

#### 2.1.2. Growth Characteristics

Strain SNU WT3 was isolated from the kidney of diseased rainbow trout from a trout farm in the Republic of Korea. The bacteria were cultured on cytophaga agar for 48 h at 25 °C and then subcultured on tryptic soy agar (TSA) (BD Difco, Detroit, MI, USA) for pure isolation, under the same conditions. Circular colonies with a convex surface and nontransparent whitish color formed on the agar. The bacteria were aerobic and psychrotolerant, as they were able to grow in temperatures ranging from 2 °C to 30 °C and were unable to grow in anaerobic conditions. The pH tolerance was also wide, ranging from 4 to 7. NaCl tolerance was estimated as up to 6% on both TSA and cytophaga agar.

#### 2.1.3. Morphology

**Figure 2:**
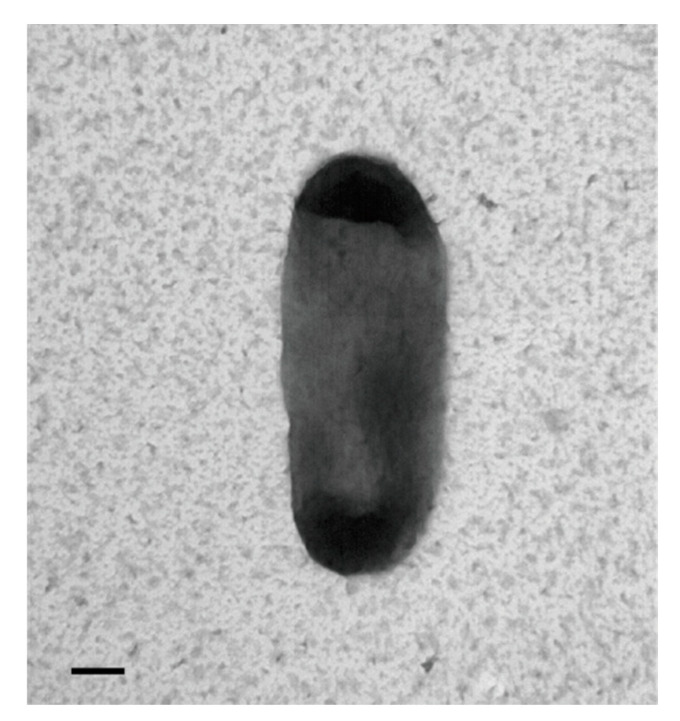
Transmission electron microscopy scanning of *Janthinobacterium tructae* SNU WT3 sp. nov. The bacteria were negatively stained with 0.5% uranyl acetate and scanned at 80 kV. Scale bar = 200 nm.

SNU WT3 bacteria were Gram-negative and had a rod-shaped appearance under the optical microscope. In the morphological analysis using transmission electron microscopy (TEM; JEM 1010, Akishima, Japan), cells of the strain SNU WT3 showed slightly curved rod shapes 1–2 µm in width and 2–3 µm in height (Figure 2).

#### 2.1.4. Molecular Configuration

The major fatty acids of strain SNU WT3 were cylco-C_17:0_ (41.45%), C_16:0_ (33.86%), and C_12:0_ (5.87%). The concentrations of major fatty acids were different from those of other *Janthinobacterium* species in the amount of cyclo-C_17:0_, which occupy the largest portion (Table 1) [8,16]. The analysis of respiratory quinones indicated that strain SNU WT3 contained only ubiquinone Q-8. The polar lipid compositions of strain SNU WT3 were phosphatidylethanolamine, phosphatidylcholine, phosphatidylglycerol, diphosphatidylglycerol, unidentified phospholipid, and an unidentified lipid.

#### 2.1.5. Biochemical Analysis

Using analytical profile index (API) CH test strips (bioMérieux, Seoul, Korea), reactions were positive for glycerol, L-arabinose, ribose, D-xylose, galactose, D-glucose, D-fructose, D-mannose, inositol, mannitol, sorbitol, esculine, cellobiose, maltose, lactose, saccharose, trehalose, xylitol, D-lyxose, and D-arabitol. The test results were negative for erythritol, D-arabinose, L-xylose, adonitol, β-methyl xyloside, L-sorbose, rhamnose, dulcitol, α methyl-D-mannoside, α methyl-D-glucoside, N-acetylglucosamine, amygdalin, arbutin, salicin, melibiose, inulin, melezitose, D-raffinose, amidon, glycogen, β gentiobiose, D-turanose, D-tagatose, D-fucose, L-fucose, L-arabitol, gluconate, 2-Keto-gluconate, 5-keto-gluconate, β-galactosidase, arginine dihydrolase, lysine decarboxylase, ornithine decarboxylase, citrate utilization, H2S production, urease, tryptophan deaminase, indole production, acetoin production, and gelatinase. In API 20NE test strips (bioMérieux), the reactions were positive for reduction of nitrates to nitrites, esculin hydrolysis, and assimilation of glucose, arabinose, mannitol, N-acetyl-glucosamine, citrate, phenyl-acetate, and cytochrome oxidase. The test was negative for indole production, glucose acidification, arginine dihydrolase, urease, gelatin hydrolysis, assimilations of mannose, maltose, gluconate, caprate, and malate (Table 2).

#### 2.1.6. DNA-DNA Hybridization Test

In DNA-DNA hybridization tests, the similarities of DNA-DNA between SNU WT3 and *J. lividum* DSM 1522^T^, and SNU WT3 and *J. svalbardensis* JA-1^T^ were 47.11% and 39.88%, respectively, which were clearly below the recommended threshold value of 70% [17].

### 2.2. Genetic Analysis

#### 2.2.1. 16S-rRNA-Based Phylogenetic Analysis

**Figure 3:**
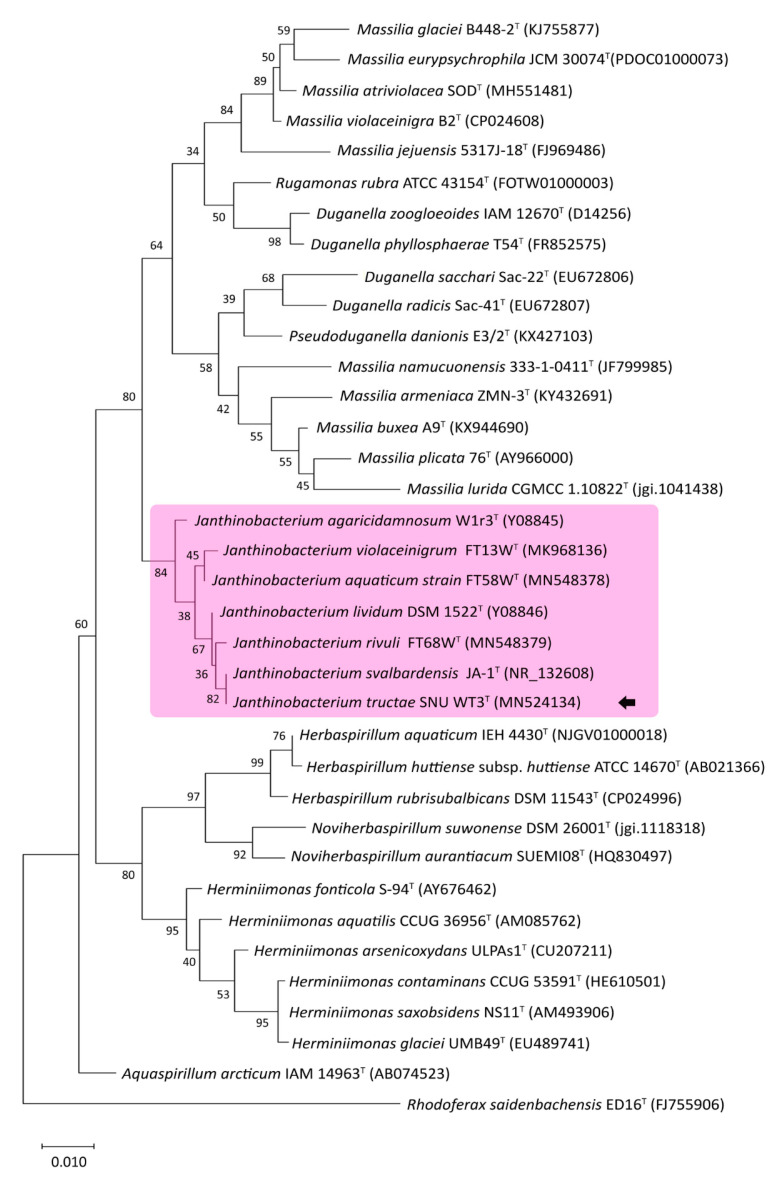
Phylogenetic tree of 16S rRNA partial sequences of *Janthinobacterium tructae* strain SNU WT3 and other related strains. An unrooted tree was constructed using the maximum likelihood method and the Tamura & Nei (1993) model with MEGA X [19,20]. The tree is drawn to scale, and the scale bar indicates 0.010 changes per nucleotide position. The maximum composite likelihood method was used to calculate evolutionary distances [21]. Missing data or gaps were completely deleted, and the phylogeny was evaluated using 1000 bootstrap replicates. The percentage values of associated taxa clustered together are presented next to the branches. *Rhodoferax saidenbachensis* strain ED16^T^ was used as an outgroup. Strain SNU WT3 is indicated with an arrow. All *Janthinobacterium* spp. are highlighted in pink. ^T^: Type strain.

As a result of EzBioCloud 16S database analysis of the 1452 base pair long 16S rRNA gene (GenBank accession number: MN524134.1) of SNU WT3, *J. svalbardensis* JA-1^T^ and *J. lividum* DSM 1522^T^ showed 100% and 99.72% similarity, respectively [18]. The phylogenetic analysis of related strains, including all *Janthinobacterium* type strains, was reported. Some of the groupings showed poor bootstrap values. However, both the nodes where *Janthinobacterium* species and other clades diverge and the strain SNU WT3 and other *Janthinobacterium* strains diverge had values over 80%, indicating reliable clustering (Figure 3). Strain SNU WT3 showed a close distance with *J. svalbardensis* JA-1^T^, *J. rivuli* FT68W^T^, and *J. lividum* DSM 1522^T^.

#### 2.2.2. Complete Genome Sequence

**Figure 4:**
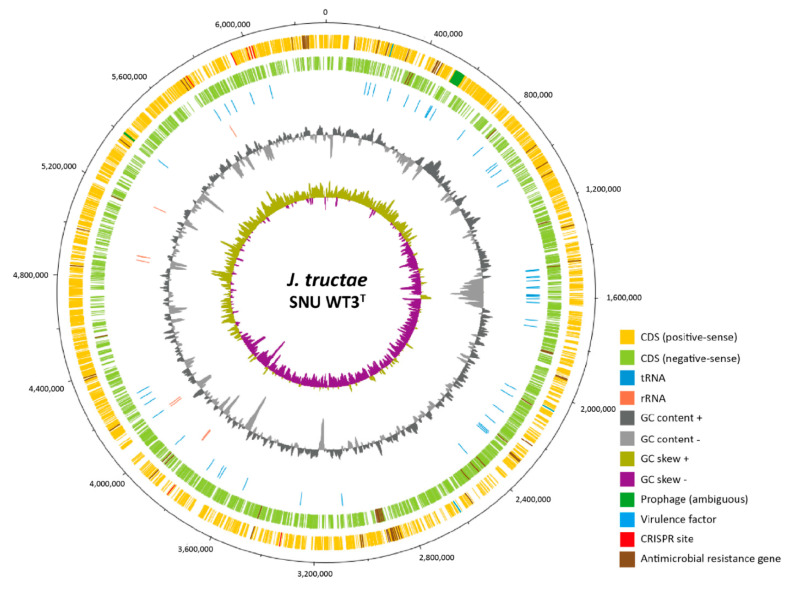
Circular plot of the complete genome of *Janthinobacterium tructae* SNU WT3^T^. The plot was drawn using DNAplotter [22]. All annotations except for tRNA and rRNA are marked on the positive and negative sense coding sequences (CDSs). tRNAs and rRNAs are marked separately from the CDSs on the third and fourth circles, respectively.

The complete genome of SNU WT3 consists of a circular chromosome of 6,314,370 bp. Hi-seq raw data consists of 13,414,480 reads with 1,354,862,480 bases. The filtered dataset showed 98.00% of the bases over Phred quality score 20 and 92.99% over Phred quality score 30. One circular contig is formed by de novo assembly, of which the total contig base number is 6,314,370, and the read depth is 136. The genome contains 5459 coding sequences (CDSs), 5403 coding genes, which produce 850 hypothetical proteins, 8 rRNAs, 93 tRNAs, and 4 ncRNAs, with a 62.35% G + C ratio. Three genes that encode THIN-B metallo-β-lactamase and a resistance-nodulation-cell division antibiotic efflux pump were detected, raising the possibility of resistance to the antibiotics beta-lactam, fluoroquinolone, and tetracycline. A total of 58 virulence factors were detected, and their gene functions are as follows: 50 related to flagella, 3 related to type IV pili, 2 related to catalase, 1 related to Zn^2+^ metalloprotease, 1 related to alternative sigma factor RpoS, 1 related to capsule I. Seven CRISPR sites and two ambiguous prophages were detected. A schematic circular plot of the complete genome, reflecting all annotations, is shown in Figure 4. The complete sequence of strain SNU WT3 was deposited in GenBank under the accession number CP041185.1.

#### 2.2.3. Average Nucleotide Identity and Genome-to-Genome Distance Calculator Analysis

**Figure 5:**
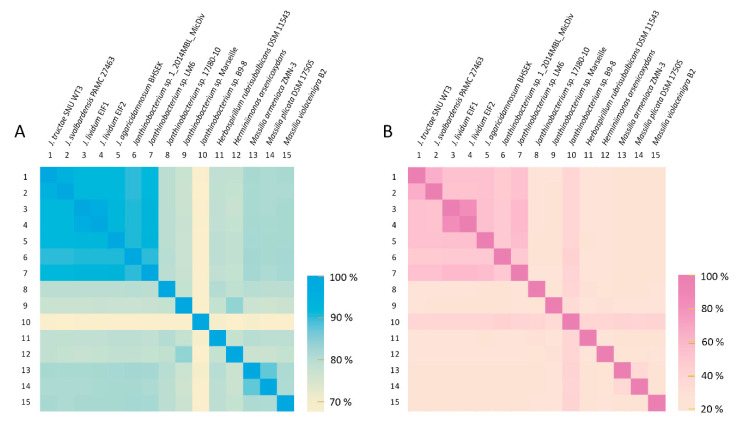
Heat map of average nucleotide identity (ANI) values and genome-to-genome distance calculator (GGDC) values compared among 15 related strains. (**A**) ANI heat map. (**B**) GGDC heat map. ANI and GGDC values are indicated by color intensity.

Of the reported complete genome data, all *Janthinobacterium* spp. strains and some other related species were used for ANI and GGDC analysis of SNU WT3, and the results indicated that strain SNU WT3 is a novel species. Based on the ANI values, *J. svalbardensis* PAMC 27463, *Janthinobacterium* sp. 17J80-10, and *J. lividum* EIF1 were the closest strains to SNU WT3, with ANI values of 95.0%, 91.8%, and 91.7%, respectively, all of which were below the cutoff value of 95% (Table 3) [23]. *J. svalbardensis* PAMC 27463, *Janthinobacterium* sp. 17J80-10, and *J. agaricidamnosum* BHSEK were the three strains with the highest GGDC values of 60.6%, 46.1%, and 45.9%, which were below the cutoff value of 70% for species differentiation [24]. To determine the correlation pattern between species and strains based on their ANI and GGDC values, a heat map was drawn (Figure 5). Some *Janthinobacterium* strains showed a relatively stronger correlation, forming a clade (Figure 5).

#### 2.2.4. Core Genome Phylogeny and Multilocus Sequence Analysis (MLSA)

**Figure 6:**
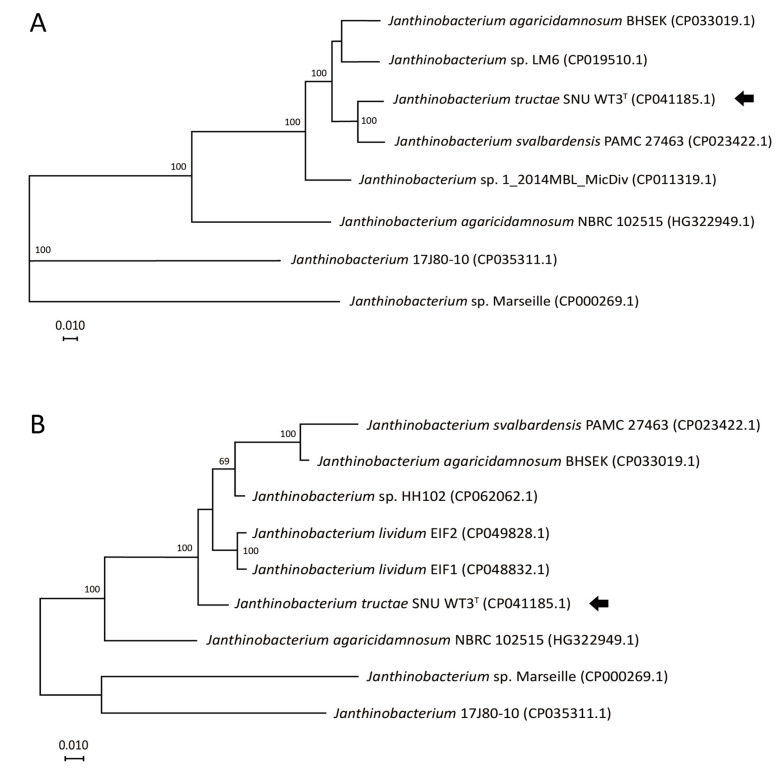
Core genome phylogeny and multilocus sequence alignment (MLSA) tree of *Janthinobacterium* species. (**A**) Core genome phylogenetic tree was constructed using EDGAR 2.0 [25]. Core gene sets were aligned using MUSCLE and concatenated. An approximately maximum-likelihood phylogenetic tree was constructed with FastTree. Local support values were shown next to the branches as percentages. (**B**) An unrooted tree was constructed from four concatenated housekeeping genes extracted from the complete genome sequences of nine *Janthinobacterium* strains. The tree was inferred using the maximum-likelihood method and the Tamura and Nei (1993) model with MEGA X and was drawn to scale [19,20]. The maximum composite likelihood method was used to calculate evolutionary distances [21]. Missing data or gaps were completely deleted, and the phylogeny was evaluated using 1000 bootstrap replicates. The percentage values of associated taxa clustered together are indicated next to the branches. Scale bars = 0.010 changes per nucleotide position. Strain SNU WT3 is indicated with an arrow.

Phylogenetic tree based on core genome showed separation between SNU WT3^T^ and *J. svalbardensis* PAMC 27463 with 100 local support values. The MLSA result using four housekeeping genes showed that the *J. svalbardensis* PAMC 27463 with the highest ANI and GGDC values (95.0% and 60.6%, respectively) had farther evolutionary distance than other *Janthinobacterium* species to SNU WT3^T^ (Figure 6). The bootstrap value of the node that SNU WT3^T^ and other *Janthinobacterium* species diverge was 100.

## 3. Discussion

The 16S rRNA sequence of strain SNU WT3 was analyzed for species identification and was identical to that of the *J. svalbardensis*. However, unlike other related *Janthinobacterium*, such as *J. svalbardensis* and *J. lividum*, strain SNU WT3 did not show purple pigmentation, which is known to be produced by violacein [6,14]. This was one of the early clues that led to the suspicion that strain SNU WT3 may be a separate species, even with the 100% identical 16S rRNA sequence with *J. svalbardensis*. *VioA*, *VioB*, *VioC*, *VioD*, *VioE*, the genes coding for violacein in *Janthinobacterium*, was not found in the complete genome of SNU WT3.

Chemotaxonomic analyses showed differences with other *Janthinobacteria*, as *J. agaricidamnosum*, *J. violaceinigrum*, *J. aquaticum*, *J. rivuli* and *J. svalbardensis* do not contain phosphatidylcholine, one of the main components of strain SNU WT3, in their polar lipid composition [5,6,16]. The concentrations of major fatty acids were different from those of other *Janthinobacterium* species in the amount of cyclo-C_17:0_, which occupied the largest portion in SNU WT3 [8,16]. Biochemical analysis showed that strain SNU WT3 could be differentiated from its most closely related strain, *J. svalbardensis* JA-1^T^, on D-xylose, adonitol, N-acetylglucosamine, arbutin, and cellobiose. The strain can also be distinguished from another close relative, *J. lividum* DSM 1522^T^, by its levels of D-arabinose, arbutin, salicin, trehalose, xylitol, L-fucose, and 2-ketogluconate [6]. DNA-DNA hybridization indicated that strain SNU WT3 does not belong to any groups of *J. lividum* or *J. svalbardensis* and is a novel species of the genus *Janthinobacterium*.

The only results that did not show distinct differences between SNU WT3 and other related strains were the respiratory quinone composition and the 16S rRNA sequence. All *Janthinobacteria* have only ubiquinone Q-8 as their respiratory quinone, and the 16S rRNA sequence of *J. svalbardensis* JA-1^T^ showed 100% identity with the strain SNU WT3 [5,6,8]. However, all other results from core genome phylogeny, MLSA, complete genome, biochemical, and chemotaxonomic analyses showed marked differences between SNU WT3 and other related strains, supporting the conclusion that SNU WT3 is a novel species. Hereby, we propose that the strain SNU WT3 is classified as a novel species of the genus *Janthinobacterium*, designated as *Janthinobacterium tructae* sp. nov. (truc’tae L. gen. n. *tructae* of a trout). The type strain *J. tructae* SNU WT3 was deposited to Korean Collection for Type Cultures and Japan Collection of Microorganisms (KCTC 72518; JCM 33613).

*Janthinobacterium tructae* strain SNU WT3^T^ was isolated from the kidney of diseased rainbow trout from a farm in the Republic of Korea. There are only a few reports of *Janthinobacterium* isolated from piscine species, describing the isolation of *J. lividum*. Both *J. tructae* strain SNU WT3 and *J. lividum* were isolated from rainbow trout, which is one of the representative cold-water fish [10,11]. *J. svalbardensis*, the nearest species in the phylogeny, was isolated from glacier ice samples from Spitsbergen island in the Svalbard archipelago. As this area is the natural habitat of the Arctic charr, which belongs to the family Salmonidae, a group that includes the rainbow trout, it is necessary to keep monitor the interactions between *Janthinobacterium* and the local piscine species [26].

## 4. Materials and Methods

Disease diagnosis of rainbow trout fingerlings (20 ± 3 g) was requested by one of the rainbow trout farms in the Republic of Korea. The fish exhibited abnormal swimming behavior, and mortality of the trout farm was 12–16%, which was higher than the usual mortality of 4%. Through postmortem examination, we verified swollen kidneys from moribund rainbow trout, and the strain SNU WT3 was isolated from the affected organ. For histopathological examination, the kidney, liver, pancreas, intestine, and pyloric cecum of moribund fish were sampled in buffered formalin. Histology slides were made and stained with hematoxylin and eosin.

For the bacterial isolation, the kidney of the moribund fish was sampled and homogenized using 300 µL PBS. The 100 µL homogenized solution was distributed on Cytophaga and TSA and incubated at 20 °C and 25 °C for 48 h. Transparent whitish colonies appeared on the Cytophaga plates, and the colonies were re-streaked for pure isolation on TSA at 25 °C for 24 h. The colonies showed uniform shape, and one of the colonies was chosen and subcultured for the analysis of the genome, biochemical details, and chemotaxonomic characteristics. The strain SNU WT3 was stored in tryptic soy broth (TSB) (BD Difco, Franklin Lakes, NJ, USA) supplemented with 25% glycerol at −80 °C.

The growth range and pH tolerance of strain SNU WT3 were tested. The bacteria were cultured on TSB at 2, 4, 10, 15, 20, 25, 37 and 45 °C. For the pH growth test, TSB medium was used and adjusted with HCl and NaOH to reach values of 4.0–11.0 at intervals of 1.0 pH unit. For the NaCl tolerance test, the test was performed in 2, 4, 6, 8, and 10% concentration in TSB, using a shaking incubator. To determine growth under anaerobic conditions, the strain was cultured in TSB medium with a paraffin blocked tube on top at 25 °C.

Analysis of the cellular fatty acid composition was performed by the Korean Culture Center of Microorganisms (KCCM) identification service, Republic of Korea. The fatty acid methyl esters were analyzed using gas chromatography in accordance with the protocol of the Sherlock Microbial Identification System (MIS, Newark, DE, USA). The profiles of cellular fatty acids were compared using the RTSBA v6.0 library database [27].

For the analysis of respiratory quinone and polar lipids, the strain was cultured in TSB at 25 °C for 24 h. It was then sent to KCCM for identification, and the strain was stained with molybdophosphoric acid and ninhydrin to show all lipids.

For the identification of biochemical details, the phenotypic characteristics of the strain were analyzed using API 20 NE and API 50 CH strips, then incubated at 25 °C for 24 h. To compare the differences in biochemical characteristics, the results were compared with data from other *Janthinobacterium* species; *J. lividum* DSM 1522^T^, *J. svalbardensis* JA-1^T^, *J. agaricidamnosum* DSM 9628 ^T^, *J. violaceinigrum* FT13W^T^, *J. aquaticum* FT58W^T^, *J. rivuli* FT68W^T^, and *J. psychrotolerans* S3-2^T^ that were analyzed in prior studies [5,6,7,8].

For morphological analysis, the strain SNU WT3 was observed using TEM at 80 kV. For negative staining, a bacterial colony cultured on TSA was suspended in PBS and negatively stained with an equal volume of 0.5% uranyl acetate. Gram staining was carried out using Gram staining Kits (bioMérieux, Seoul, Korea).

Total genomic DNA of the strain SNU WT3 was extracted from pure colonies cultured on TSA. The colonies were suspended in 300 µL Tris-EDTA buffer, heated at 100 °C for 20 min, and centrifuged at 8000 g for 10 min. After centrifugation, the pellet was discarded, and 100 µL of remaining supernatant was used for polymerase chain reaction (PCR) of the 16S rRNA gene. For gene sequencing, universal primers (24 F, 1492R) were used [28,29]. The final PCR product was sequenced using an ABI PRISM 3730XL Analyzer with BigDye^®^ Terminator v3.1 cycle sequencing kits (Applied Biosystems, Foster, CA, USA) by Macrogen inc. (Seoul, Korea).

For phylogenetic analysis, the partial sequence of the retrieved 16S rRNA gene was used. The alignment of the sequences was edited using BioEdit software, and the sequence of the gene from strain SNU WT3 was compared to those of other strains using NCBI BLAST and the EzBioCloud server for the identification of subspecies [30,31,32]. The phylogenetic tree was constructed using a maximum-likelihood method, using MEGA X software, and the genetic distances were estimated using a Tamura and Nei (1993) model [19,20]. The topology of the tree was assessed using bootstrap analysis with 1000 replicates.

DNA-DNA hybridization analysis was carried out by KCCM to confirm the differences, indicating that strain SNU WT3 was a novel species. The strains used for the comparative analysis were *J. svalbardensis* JA-1^T^ and *J. lividum* DSM 1522^T^. *J. svalbardensis* JA-1^T^ was identified as the closest species by the phylogenetic analysis, followed by *J. lividum* DSM 1522^T^ and *J. rivuli* FT68W^T^.

The complete genome sequencing of the strain SNU WT3 was performed by Macrogen, Inc. (Seoul, Republic of Korea) using a hybrid approach with a PacBio RS II system (Pacific Biosciences, Menlo Park, CA, USA) on the HiSeq 2000 platform (Illumina, San Diego, CA, USA). Hierarchical Genome Assembly Process (HGAP) v. 3.0 was used for genome de novo assembly of data with 90.82× coverage [33]. Genome annotation was performed using the NCBI’s Prokaryotic Genome Annotation Pipeline with the Best-placed reference protein set and GeneMarkS-2+ [34,35]. Antibiotic resistance genes were detected using ARG-ANNOT and Resistance Gene Identifier, with the Comprehensive Antibiotic Research Database, and virulence factors were identified with VFanalyzer [36,37,38]. Prophages were detected using Prophage Hunter, and CRISPR loci were detected using the CRISPR Recognition Tool [39,40]. The schematic structure of the complete genome was drawn using DNAPlotter [21]. The genome of strain SNU WT3 was compared to those of related strains, including all *Janthinobacterium* species with complete genome data available, using the ANI calculator of the OrthoANIu tool [41], and the intergenomic distance calculator by GGDC v2.1 (DSMZ) formula 2 [42,43,44]. Core genome phylogeny was analyzed using EDGAR 2.0 [25]. Core genes of *Janthinobacterium* with complete genome sequences were computed, aligned using MUSCLE, and concatenated for approximately maximum-likelihood phylogenetic tree construction in the FastTree software (http://www.microbesonline.org/fasttree/ accessed on 8 February 2021). For MLSA, concatenated sequences were generated by extracting four housekeeping genes (aroE, gyrB, RecA, rpoB) that were used for MLSA in *Janthinobacterium* in a previous study [13]. Phylogenetic tree for MLSA was constructed using the same method that was used for drawing the tree based on 16S rRNA gene sequence.

## Figures and Tables

**Table 1:** Compositions of cellular fatty acid contents (%) of strain SNU WT3 and six reference strains.

Fatty Acid	*J. tructae* SNU WT3^T^	*J. lividum* CCUG2344^T^ [16]	*J. svalbardensis* JA-1^T^ [8]	*J. agaricidamnosum* CCUG43104^T^ [16]	*J. violaceinigrum* FT13W^T^ [8]	*J. aquaticum* FT58W^T^ [8]	*J. rivuli* FT68W^T^ [8]
cylco-C_17:0_	41.45	25.0	ND	34.2	ND	ND	ND
C_16:0_	33.86	30.6	33.9	34.8	31.3	29.3	35.1
C_12:0_	5.87	3.9	3.8	3.0	8.1	5.6	6.2

ND: not detected, ^T^: Type strain.

**Table 2:** Biochemical details compared between strain SNU WT3 and its close relatives identified using API 20NE and API 50CH kits.

	*J. tructae* SNU WT3^T^	*J. lividum* DSM 1522^T^ [6]	*J. svalbardensis* JA-1^T^ [6]	*J. agaricidamnosum* DSM 9628 ^T^ [6]	*J. violaceinigrum* FT13W^T^ [8]	*J. aquaticum* FT58W^T^ [8]	*J. rivuli* FT68W^T^ [8]	*J. psychrotolerans* S3-2^T^ [7]
D-arabinose	−	+	−	−	−	+	+	−
L-arabinose	+	+	+	−	+	+	W	+
D-xylose	+	+	−	−	+	+	+	NP
Adonitol	−	−	+	−	NP	NP	NP	NP
Galactose	+	+	+	−	−	+	+	+
Sorbitol	+	+	+	−	−	+	+	−
N-acetylglucosamine	−	−	+	−	NP	NP	NP	−
Arbutin	−	+	+	−	NP	NP	NP	NP
Salicin	−	+	−	−	NP	NP	NP	+
Cellobiose	+	+	−	−	−	+	−	+
Maltose	+	+	+	−	−	−	+	−
Trehalose	+	−	+	+	−	−	-	−
Xylitol	+	−	+	−	NP	NP	NP	NP
β gentiobiose	−	−	−	+	NP	NP	NP	NP
D-lyxose	+	+	+	−	−	+	-	NP
L-fucose	−	+	−	−	−	+	+	+
2-ketogluconate	−	+	−	−	NP	NP	NP	+
Rhamnose	−	−	−	−	NP	NP	NP	+
Inulin	−	−	−	−	NP	NP	NP	NP
D-raffinose	−	−	−	−	NP	NP	NP	+

NP: not performed, W: weakly positive, ^T^: Type strain, −: Negative, +: Positive.

**Table 3:** Average nucleotide identity (ANI) values and genome-to-genome distance calculator (GGDC) values between strain SNU WT3 and other related species.

	*Janthinobacterium tructae* SNU WT3 (ANI Value)	*Janthinobacterium tructae* SNU WT3 (GGDC Value)
*Janthinobacterium svalbardensis* PAMC 27463	95.0%	60.6%
*Janthinobacterium lividum* EIF1	91.7%	45.7%
*Janthinobacterium lividum* EIF2	91.7%	45.7%
*Janthinobacterium agaricidamnosum* BHSEK	91.7%	45.9%
*Janthinobacterium* sp. 1_2014MBL_MicDiv	89.4%	38.5%
*Janthinobacterium* sp. 17J80-10	91.8%	46.1%
*Janthinobacterium* sp. B9-8	67.1%	20.3%
*Janthinobacterium* sp. LM6	74.5%	20.6%
*Janthinobacterium* sp. Marseille	73.0%	31%
*Herbaspirillum rubrisubalbicans* DSM 11543	74.0%	20.6%
*Herminiimonas arsenicoxydans*	73.6%	20.3%
*Massilia armeniaca* ZMN-3	76.6%	21.0%
*Massilia plicata* DSM 17505	76.3%	21.1%
*Massilia violaceinigra* B2	76.6%	21.3%

## Data Availability

Data is contained within the article.
